# Thermographic Profiles in Livestock Systems under Full Sun and Shaded Pastures during an Extreme Climate Event in the Eastern Amazon, Brazil: *El Niño* of 2023

**DOI:** 10.3390/ani14060855

**Published:** 2024-03-11

**Authors:** Welligton Conceição da Silva, Jamile Andréa Rodrigues da Silva, Lucieta Guerreiro Martorano, Éder Bruno Rebelo da Silva, Carlos Eduardo Lima Sousa, Kedson Alessandri Lobo Neves, Cláudio Vieira de Araújo, Leonel António Joaquim, Thomaz Cyro Guimarães de Carvalho Rodrigues, Tatiane Silva Belo, Raimundo Nonato Colares Camargo-Júnior, José de Brito Lourenço-Júnior

**Affiliations:** 1 Postgraduate Program in Animal Science (PPGCAN), Institute of Veterinary Medicine, Federal University of Para (UFPA), Castanhal 68746-360, PA, Brazil; eder.b.rebelo@gmail.com (É.B.R.d.S.); jleokim.a@hotmail.com (L.A.J.); thomazguimaraes@yahoo.com.br (T.C.G.d.C.R.); camargojunior@gmail.com (R.N.C.C.-J.); joselourencojr@yahoo.com.br (J.d.B.L.-J.); 2 Institute of Animal Health and Production, Federal Rural University of the Amazon (UFRA), Belem 66077-580, PA, Brazil; jamileandrea@yahoo.com.br; 3 Embrapa Eastern Amazon, Santarem 68010-180, PA, Brazil; lucieta.martorano@embrapa.br; 4 Department of Veterinary Medicine, University Center of the Amazon (UNAMA), Santarem 68010-200, PA, Brazil; cadu34.medvet@gmail.com (C.E.L.S.); tatianebelovet@gmail.com (T.S.B.); 5 Institute of Animal Science, Federal University of Western Pará (UFOPA), Santarem 68040-255, PA, Brazil; kedson_neves@hotmail.com; 6 Department of Agricultural and Environmental Sciences, Federal University of Mato Grosso (UFMT), Sinop 78550-728, MT, Brazil; cvaufmt@gmail.com

**Keywords:** grazing areas, thermal images, shade cover, Eastern Amazon region

## Abstract

**Simple Summary:**

The *El Niño* presents itself as a serious problem in the pastures of the northern region of Brazil, as it compromises the availability and quality of forage and water. Therefore, the objective of this study was to characterize the thermographic profile of three production systems in the Eastern Amazon, Brazil. The results show significant differences between areas with and without chestnut tree shade. Between August and November, the highest temperatures were recorded in full sun pastures, contrasting with lower temperatures in shaded areas. The interaction between the systems revealed significant thermal variations, highlighting the positive impact of native trees on thermal regulation and indicating possible strategies to mitigate the adverse effects of *El Niño*.

**Abstract:**

The *El Niño* represents a substantial threat to pastures, affecting the availability of water, forage and compromising the sustainability of grazing areas, especially in the northern region of Brazil. Therefore, the objective of this study was to characterize the thermographic profile of three production systems in the Eastern Amazon, Brazil. The study was conducted on a rural cattle farm in Mojuí dos Campos, Pará, Brazil, between August and November 2023. The experiment involved livestock production systems, including traditional, silvopastoral and integrated, with different conditions of shade and access to the bathing area. An infrared thermographic (IRT) camera was used, recording temperatures in different zones, such as areas with trees, pastures with forage and exposed pastures. The highest mean temperatures (*p* = 0.02) were observed in pastures with full sun from August to November. On the other hand, the lowest average temperatures were recorded in areas shaded by chestnut trees (*Bertholletia excelsa*). The highest temperature ranges were found in sunny areas and the lowest were recorded in shaded areas. The highest temperatures were observed in the pasture in full sun, while the lowest were recorded in areas shaded by chestnut trees (*p* < 0.0001). The interaction between the systems and treatments revealed significant temperature differences (*p* < 0.0001), with the native trees showing an average temperature of 35.9 °C, lower than the grasses and soil, which reached 61.2 °C. This research concludes that, under *El Niño* in the Eastern Amazon, areas shaded by Brazil nut trees had lower temperatures, demonstrating the effectiveness of shade. Native trees, compared to grasses and soil, showed the ability to create cooler environments, highlighting the positive influence on different species such as sheep, goats and cattle.

## 1. Introduction

Three systems of cattle farming can be found in the Eastern Amazon: traditional, which is the one most cattle farmers use, with no trees or bathing places for the animals; integrated, with shade and water for bathing; and silvopastoral, with shade from trees but no water for bathing. Thus, systems where the animals have access to water from existing rivers and lakes to cool off during periods of intense solar radiation and areas with pastures shaded by native tree species are important to ensure the animals’ thermal comfort during the period of highest solar radiation. However, there are still systems where pastures are predominantly in full sun, with access to water only at drinking fountains [1,2,3]. A major scientific concern is reconciling animal welfare with productive performance in Brazilian livestock farming [4,5,6,7].

Exposure to direct sunlight makes animals susceptible to heat stress. Heat stress has a negative impact on the animal’s physiology and productivity, as the animal’s heat dissipation capacity can be exceeded [8,9]. Therefore, animal welfare (AW) advocates the combination of environment and productivity, in which behavioral reactions can be observed in animals, depending on where they are, influencing production and economic impacts [6,10,11,12,13,14].

Even considering that cattle have thermoregulatory mechanisms that help dissipate accumulated internal heat, such as increased vasodilation, respiratory rate, heart rate, body and rectal temperature, as well as frequent urination and defecation, it is necessary to observe the limiting conditions in terms of extreme thermal values, especially in regions with high temperatures, as is the case in regions in the equatorial belt. Research shows that high or very low temperatures tend to hinder thermoregulation, leading to various physiological reflexes, such as increased heart and respiratory rates, sialorrhea and metabolic or reproductive problems, such as an increase in cortisol, glucose and a reduction in the animals’ digestion process, as well as a reduction in the quality of female oocytes and spermatogenesis, causing losses. In this sense, cattle regulate the excess solar radiation that comes into direct contact with their bodies by seeking shade, shelter or water to mitigate heat stress [15,16,17,18,19,20,21,22].

Among the technologies used to investigate animal comfort and environmental conditions quickly, non-invasively and with reliable and replicable results, monitoring using near-infrared thermography has been gaining ground, with applications in different areas of scientific knowledge, such as early diagnosis of diseases, non-invasive assessment of rectal temperature, determination of animal thermal stress and thermal characterization of the environment in which they live [23,24]. The radiometric imaging allow data to be obtained by evaluating targets such as the surface of animals and correlating them with the production systems studied [25,26,27,28,29,30].

Thus, characterizing the thermographic profile of different animal production systems in the Brazilian Amazon becomes important due to the region’s peculiar environmental conditions. The Amazon faces intense climatic challenges, such as high temperatures and humidity, which can have a significant impact on animal welfare and performance. By evaluating the thermal variations of the environment in which the animals live, thermography allows for a more in-depth understanding of the thermal conditions of production systems, which makes it possible to adopt management strategies to improve the productive efficiency and health of the herds in each system [28,31,32,33,34,35].

Some factors are beyond human control, such as years when extreme climatic phenomena occur, for example, the effects of *El Niño*/Southern Oscillation years, which in the Amazon have been associated with an intensification of the period of reduced rainfall in the region, causing periods of water scarcity in the soil and an increase in air and soil temperature. This adverse climate pattern represents a significant threat to pastures and, consequently, to the animals that depend on these grazing areas to guarantee the daily food supply of their livestock [36,37]. Therefore, rising temperatures and decreasing rainfall during *El Niño* events contribute to a water deficit in pastures, reducing the quantity and quality of plants available for animal grazing. In addition, water scarcity and reduced forage availability directly compromise pasture capacity, further increasing animal nutrition and health problems [38,39,40,41]. Water stress can also cause soil degradation, making it more susceptible to erosion. In addition, the lack of water and feed in pastures during periods of *El Niño* can force farmers to resort to alternative practices [42,43,44,45].

Furthermore, understanding how animal production systems interact with the environment, it is possible to develop more sustainable practices and ensure more efficient production. Thus, integrating thermographic data into cattle management contributes to developing regional production systems. Furthermore, this study is a first in the thermal characterization of pastures during *El Niño* in the Eastern Amazon. The hypothesis in this study was that the environment during the period of the extreme weather event would be challenging and high in temperature and that the trees would be lower in temperature, providing a suitable environment for the animals. Therefore, this study aimed to characterize the thermographic profile of three different production systems in the Eastern Amazon, Brazil.

## 2. Materials and Methods

### 2.1. Location of the Study Area and Evaluation Period

The experiment was carried out in the municipality of Mojuí dos Campos, Pará State, Brazil, on a farm, during the rainy season (December–May) and the least rainy period (June–November) and during the transition periods of the year (from the rainiest to the least rainy—June/July and from the least rainy to the rainiest—December/January). The thermal profile presented in this work refers to the period between August and November 2023. It is worth noting that even in the wettest regions of the state of Pará, in *El Niño* years, from August onwards, estimates using the water balance considering the dominant root zone in pastures already indicate water deficits in the soil [46,47]. The months of October to November are considered the months with the highest heat index in different regions, including the north of the country [35].

**Figure 1:**
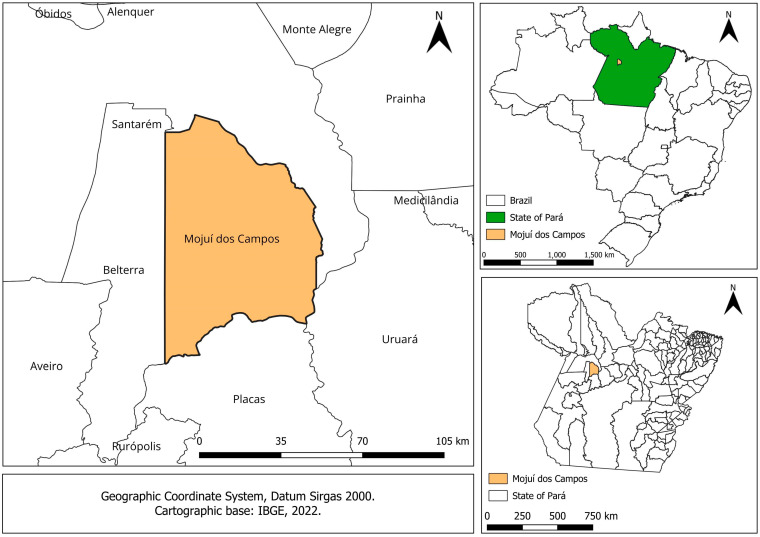
The location of the municipality where the experiment was carried out in the Eastern Amazon, Brazil.

Figure 1 shows the location map of the study area, which was carried out in an area with livestock in the municipality of Mojuí dos Campos (average altitude of 127 m, latitude 02°10′17″ S and longitude 56°44′42″ W), in the west of the state of Pará, which is part of the Eastern Amazon production hub.

### 2.2. Climate Information for the Region

The climate in the region is humid, with rainfall totals of less than 60 mm in the less rainy months and rainfall ranging from 1900 to 2100 mm, in conditions governed by the Am4 typology, in which the average annual air temperature is around 25.6 °C and the relative humidity varies from 84 to 86% [46]. In the mesoregion, the wettest quarter occurs between the months of February and April and the least rainy between August and October [47].

### 2.3. Animals and Production Systems

The study was carried out with 30 cattle weighing 250 ± 36 kg of the white Nelore breed, aged between 18 and 20 months, all uncastrated males, classified as clinically healthy, divided into three farming systems, with an ideal body score of 3.5, on a scale of 1 to 5. On the property with a livestock system, the forage *Brachiaria brizantha* cv. Marandú was planted and managed in rotational grazing with 14 days of occupation and 28 days of rest per paddock. When the pasture was planted, the necessary nutrients were applied for the two periods of the year, corresponding to the rainiest and least rainy, according to the soil analysis.

The production systems used in this study were classified as a traditional system (TS) when there were no trees in the paddocks, but they had free access to water for consumption, while in the silvopastoral system (SP) the animals were in an environment with shade from trees and access to water for consumption, while in the integrated system (IS) there was access to shade from trees, water for consumption and also for bathing.

The TS was composed only of *Brachiaria brizantha* cv. Marandú forage grass, with no trees or areas of shade. In the SP and IS, the paddocks also had the same forage plant cover, with the presence of trees native to the Amazon, such as Brazil nut trees (*Bertholletia excelsa*), in both groups. However, the animals in the SP only had water to drink, while in the IS the animals had water for both bathing and drinking. In addition, salt was offered ad libitum in all the TS, SP and IS systems. The total experimental area was 15.3 ha of *Brachiaria brizantha* cv. Marandú is divided into nine 1.7 ha paddocks, three per treatment.

### 2.4. Infrared Thermography

The data were recorded using an IRT camera [48], with emissivity set at 0.95. The camera used has a fixed lens of 25 mm, temperature range from −40 to 150 °C, sensitivity of 50 mk (>0.05 °C ambient temperature of 30 °C), spectral range from 0.7 to 100 μm, but the photographed targets have a response between 0.7 and 3.0 μm with an optical resolution of 640 × 480 pixels with a maximum emissivity index of 0.9. Temperature and humidity were controlled by positioning against the wind and the observer was positioned against the sun so as not to interfere with the camera. The thermograms were then examined using Flir Tools software, version 6.3, with the Rainbow HC palette chosen. The operator used the camera at eye level, always maintaining consistency and without the aid of a tripod, focusing perpendicular to the target, following the methodological guidelines proposed by Barreto et al. [49] and Silva et al. [50].

**Figure 2:**
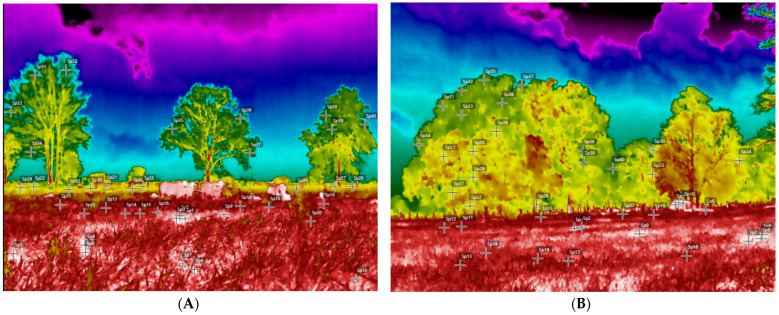

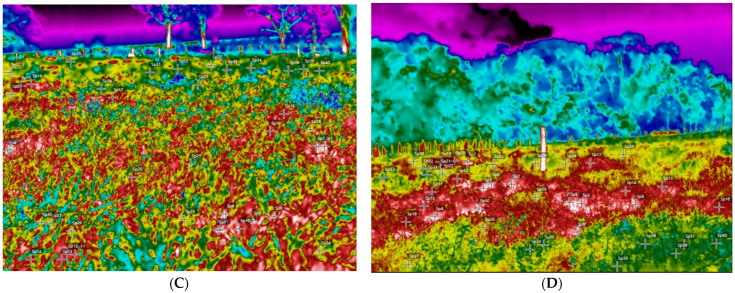
Thermographic images of the shaded paddock. (**A**) Area of ground and trees imaged. (**B**) Full sun areas imaged. (**C**) Soil exposed in a sunny area. (**D**) Exposed soil and forage.

For each zone assessed, eight radiometric images were analyzed for soil temperatures in areas with trees, pastures with forage and exposed pastures. For each thermogram and for and each color (T1—white, T2—red, T3—yellow and T4—green), 10 thermographic points were imaged (Figure 2A–D). The images were captured between 12:30 p.m. and 1:30 p.m. in August and November 2023, corresponding to the dry season in the region, which was affected by a strong *El Niño* event. In August and September, soil water deficits can be seen, even in places with higher rainfall (climate typology Af2), such as Belém. In this region, soil water deficits begin in August and persist until November, with October being the month of greatest water scarcity. Because of this radiometric images were taken in this region in September and October to highlight and monitor thermal responses in dry soil in the face of climate changes observed during the *El Niño* climate event.

### 2.5. Statistical Analysis

To verify the homogeneity of the variance of the residuals, the Bartlett’s test was used and the Shapiro–Wilk test was used to evaluate the normality of these residuals. In both cases, the hypothesis of nullity was accepted. The temperature was thus considered a continuous random variable with a normal distribution and treatments showed homogeneity of variance and were analyzed in an entirely randomized design and a subdivided plot structure, according to the line-air model:

Yijk=μ+α_i+D_((i)k)+β_j+(αβ)_ij+ε_((ij)k)

where Yijk is the vector of temperature observations in the i-th system, in the j-th treatment of the k-th repetition, the term “μ” is a general constant inherent to all observations in Yijk; α_i represents the systems (i = 1 and 2); the term D_((i)k) re-presents the plot error, characterized by the “k” repetitions nested in the “i” levels of systems, with k = 10 animals; β_j represents the effects of the treatments arranged in the subplot, with j = 1, 2, 3 and 4 (T1 = white color; T2 = red color; T3 = yellow color; T4 = green color); the term (αβ)_ij refers to the interaction between the i-th level of systems with the j-th level of treatments and is the error associated with the subplot that received level i of systems and level j of the treatment factor in repetition k, with ~NIID(0,σ_e^2).

The Tukey’s mean comparison test was used to compare levels of treatment effects, with a significance level of 0.05. The Package Statistical Analysis System [51] was used for the statistical analysis.

## 3. Results

**Figure 3:**
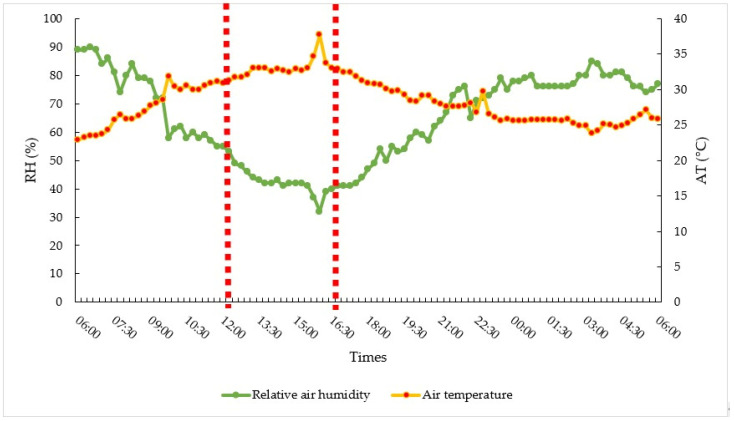
Weather data averaged from August to November 2023. Vertically dotted red bars show the time the thermographic images were taken.

The climate parameters assessed were air temperature (TA °C) and relative humidity (RH %), from the agrometeorological mini-stations set up in the experimental area. Figure 3 illustrates the agrometeorological conditions, expressed by air temperature and humidity, in the study area, showing the effect of the *El Niño* severe weather event. During this period, the temperature reached 37.7 °C at the time of highest heat load.

**Figure 4:**
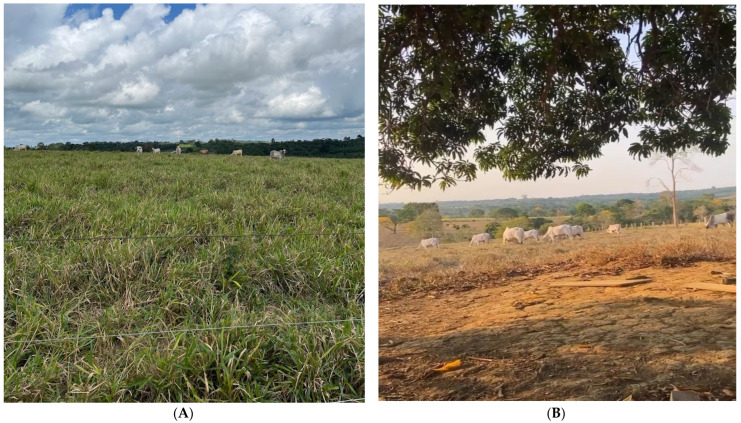

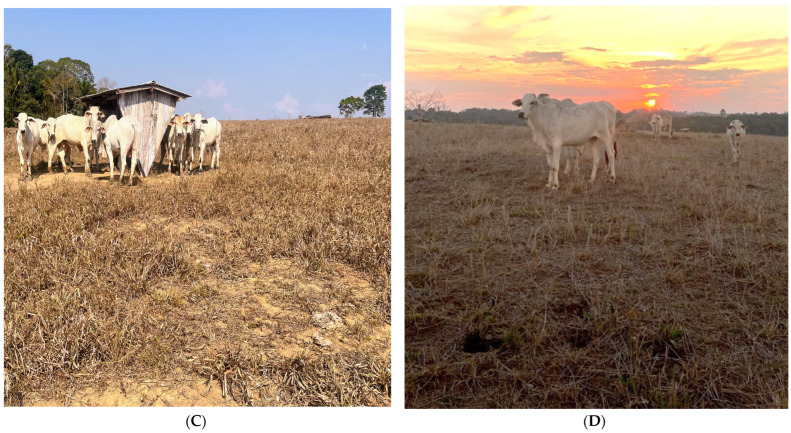
Images of the pasture area according to the months of the year during *El Niño*. (**A**) August. (**B**) September. (**C**) October. (**D**) November.

Figure 4A–D shows the evolution of the pasture during the collection of thermographic data over the months. It is possible to see the pasture with a water deficit as the months go by, and the soil becoming exposed due to the death of the forage plants, which is very evident from September 2023 onwards.

Table 1 shows the extreme temperature values (minimum and maximum), average temperature and descriptive analysis of the SH and SN environments. From August to November, the highest average temperatures were observed in the full sun pasture (*Brachiaria brizantha* cv. Marandú) (*p* = 0.02). On the other hand, the lowest average temperatures were recorded in areas shaded by chestnut trees (*Bertholletia excelsa*). The highest temperature ranges were found in sunny areas and the lowest in shaded areas.

**Figure 5:**
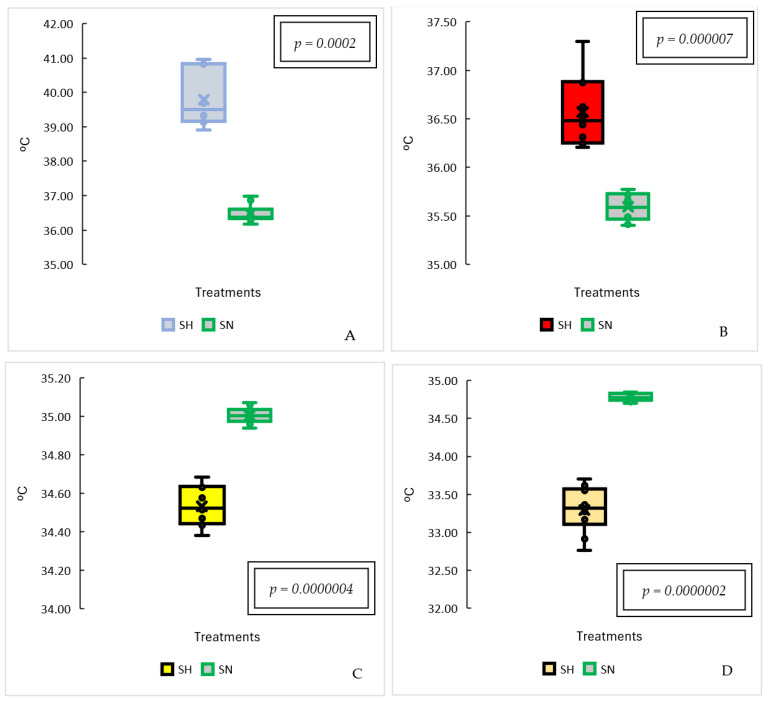
Estimated means and standard deviations for temperature in each combination of system levels and treatments. (**A**) white color standard. (**B**) red color standard. (**C**) yellow color standard. (**D**) green color standard. SH = Shadow, SN = Sun.

There were significant differences between the treatments in full sun and under the shade of the Brazil nut tree (*Bertholletia excelsa*) for the thermal responses at the imaged points (Figure 5). For the sun and shade treatments where the temperatures were higher, within the lighter-colored thermal patterns (Figure 5A,B), it can be seen that the temperatures in the pasture area in full sun were higher than those in the shade (*p* < 0.0001). However, it was possible to observe that the darker color patterns showed lower temperatures in both treatments (Figure 5C,D) (*p* < 0.0001).

There was a significant interaction effect between the effects of systems and treatments (*p* < 0.0001). In the sun system, the highest temperature values were found for treatments T1 and T2, respectively, with treatments T3 and T4 showing similar temperatures to each other and lower than the other treatments (Table 2). In the chestnut tree (*Bertholletia excelsa*) shade system, all the treatments differed in temperature, with higher average values for T1, T2, T2 and T4, in that respective order.

**Figure 6:**
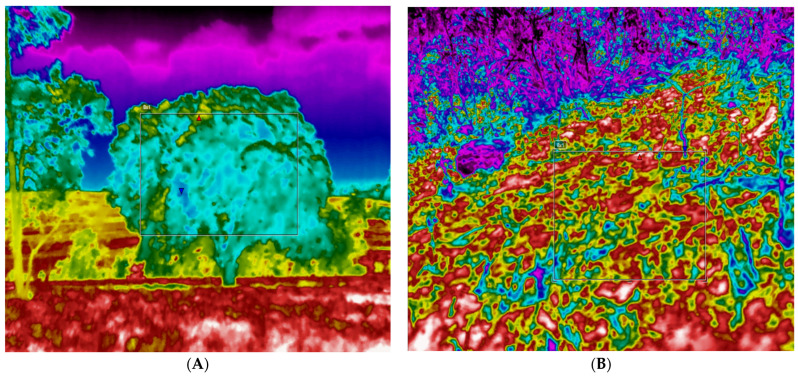

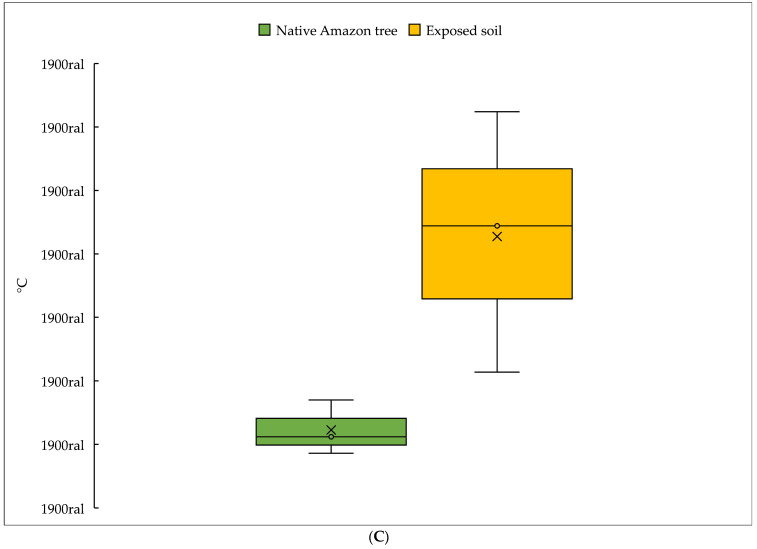
Thermal images and box plots of the minimum, maximum and average temperatures evaluated. (**A**) native Amazon tree. (**B**) exposed soil. (**C**) Averages for exposed soil (yellow) and shaded areas (green).

The average temperature of the native trees in the Amazon region was 35.9 °C, 25.3 °C lower than the temperature of the grasses and soil, which reached 61.2 °C (Figure 6) (*p* < 0.05).

## 4. Discussion

The temperatures recorded in the thermal patterns in this study demonstrate a challenging scenario for raising beef cattle in a tropical region, being outside the range considered ideal by Baêta and Souza [52], who established favorable climatic conditions between 10 and 27 °C. The constant increase in temperatures indicates the need for innovative approaches to tackling the thermal challenges associated with livestock farming in this environment.

The study highlights the role of shading provided by trees in silvopastoral systems as an effective strategy to mitigate the adverse impacts of heat, thus improving the thermal comfort of animals, especially during the hottest periods of the day. Thus, trees in pasture areas are important to help with the thermo-regulation of different species of animals, such as cattle and sheep, as the trees block the passage of the sun’s rays, making the environment cooler and more comfortable, reducing heat stress and improving the well-being and health of the animals. In addition, the use of infrared thermography in the silvopastoral system in the western region of the state of Pará offers a more detailed perspective of the thermal variations in the environment during *El Niño*.

Numerous studies have highlighted the relationships between microclimate variables and canopy thermal characteristics in pasture environments [35,53,54,55,56,57,58]. Do not consider these interactions to influence substantial errors in climate measurements, due to temperature variation between the ground surface and the air masses above the canopy [59]. Thus, this study provides direct evidence of climate change, as it presents the effects of *El Niño* in the Amazon region, as well as its repercussions for the region’s ecosystem [60,61,62], especially the low availability of pasture as a source of food for animals.

This study is a pioneer in the characterization of infrared thermal patterns during *El Niño* in the Amazon region. The evolution of the pasture observed during the collection of thermographic data over the months during the *El Niño* climatic event reveals the presence of a water deficit, especially from September onwards, resulting in the exposure of the soil due to the death of the forage plants. This can be explained because in Brazil, *El Niño* has been associated with increased risks of drought and higher temperatures [63,64,65,66,67], especially in the northern regions of the country.

The adverse weather pattern presented by *El Niño* poses a significant threat to pastures and, consequently, to the animals that depend on these areas for food. The high temperatures and decreased rainfall during *El Niño* contribute to a water deficit in pastures, resulting in a reduction in the quantity and quality of plants available for animal grazing [68,69,70]. Thus, the concomitant reduction in precipitation and increase in atmospheric vapour pressure deficit (VPD) has led to increased tree mortality [71,72,73,74,75,76].

This condition, coupled with water scarcity and reduced forage availability, directly compromises the ability of pastures to support animals, leading to nutrition and health problems. In addition, water stress can cause soil degradation, making it more susceptible to erosion. In addition, the lack of water and feed in pastures during *El Niño* periods can force farmers to resort to alternative practices, which can have significant economic and social impacts [77,78].

The data presented in Table 1 reveal distinct patterns in thermal variations between sunny and shaded environment from August to November. The highest average temperatures were recorded in the full sun pasture (*Brachiaria brizantha* cv. Marandú). This result is in line with the scientific understanding that areas directly exposed to solar radiation tend to experience higher temperatures due to the direct absorption of solar energy. The increase in temperature results in a greater thermal load on the environment, which consequently translates into an intensification in the emission of infrared radiation [28,79].

On the other hand, areas shaded by chestnut trees (*Bertholletia excelsa*) had the lowest was recorded average temperatures. The presence of trees or shade structures plays a crucial role in local thermal regulation, providing a cooler environment. Shade reduces direct exposure to the sun’s rays, minimizing heat absorption and therefore resulting in milder temperatures, as observed in the data by Silva et al. [6].

The results indicate that the highest thermal amplitudes were found in areas with direct exposure to the sun, while the lowest were recorded in shaded areas. This observation is in line with meteorological principles, as environments more exposed to the sun tend to experience a wide variation in temperatures between day and night due to the greater absorption and release of heat.

The thermal oscillations identified between the environments indicate the ability of the area with the presence of vegetation to alter the environment and its thermal conditions, as this has a direct impact on the incidence of solar radiation. The hottest areas were in places with no vegetation cover, indicating that these areas tend to absorb more thermal radiation, which raises the temperature of these areas, affecting the physiology of the grasses, water availability and the ecology of the environment.

The shade areas proved to be effective in reducing solar radiation, corroborating the previous findings by Lopes et al. [80] and Oliveira et al. [81], who observed thermal comfort in integrated crop-livestock-forest (ICLF) systems due to the reduction of solar radiation provided by the presence of trees. The density and spatial arrangement of trees in integrated pasture production systems induce significant microclimatic changes, even at a distance of 50 m from the trees [81,82,83,84].

The average temperature of 35.9 °C in the native trees represents a more moderate condition compared to the extremely high temperature of 61.2 °C recorded in the soil and grasses. This difference can be explained by the shading phenomenon provided by the treetops. The presence of trees in the Amazon region creates a cooler environment and attenuates direct exposure to the sun, limiting the amount of solar radiation that reaches the ground [35]. In addition, the transpiration from the trees helps to cool the environment, maintaining milder temperatures in the surroundings.

The temperature reductions identified in this study exceeded those observed by Barreto et al. [49], where the maximum reduction was 3.7% in the treatment with trees on pasture.

According to Silva [85], in pastures with the presence of trees, a direct impact was observed on the energy balance of the system, making it possible to reduce up to 80% of the incident solar radiation and 30% of the thermal load on the animals. Ref. [49], in Mato Grosso do Sul, Brazil, the presence of native trees reduced thermal load by 24.0% in July, 16.3% in August and 29.6% in September. In the same study, an integrated system with eucalyptus shade reduced thermal load by 17.0% in July, 4.6% in August and 7.5% in September. Ref. [86] indicated that at noon the reduction of the thermal load was 17.8% in the silvopastoral system compared to another treatment without the presence of shading.

It should be noted that the behavioral parameters of grazing cattle were not assessed in this study, but it was possible to observe in the field that in the hottest hours these animals seek shaded areas, a fact reported in the study by [23,80,87,88,89]. In general, animals look for areas with the presence of shadows during times of greater incidence of solar radiation since they are more susceptible to heat stress. The implementation of trees in pastures has become a sustainable option [90], due to improved welfare for livestock in tropical regions [28,50,91].

In addition to the benefit of shade for animals, silvopastoral systems have the potential to contribute to reducing greenhouse gas emissions by removing carbon from the atmosphere and storing biomass in the soil [92]. In addition, trees can become an extra source of income for landowners [93]. In a study involving buffalo, Garcia et al. [94] and Brcko et al. [3] pointed out that the inclusion of trees in tropical pastures promoted greater thermal comfort for the animals.

In addition, the shading of trees used in silvopastoral systems provides numerous benefits for both the animals and the environment, as it reduces greenhouse gas emissions and removes carbon from the atmosphere by storing it in the soil [92]. Thus, trees can become a source of income for landowners in an additional way [93]. Ref. [94] and Brcko et al. [3], in a study with buffaloes, observed that using trees in tropical pastures promoted greater thermal comfort.

**Figure 7:**
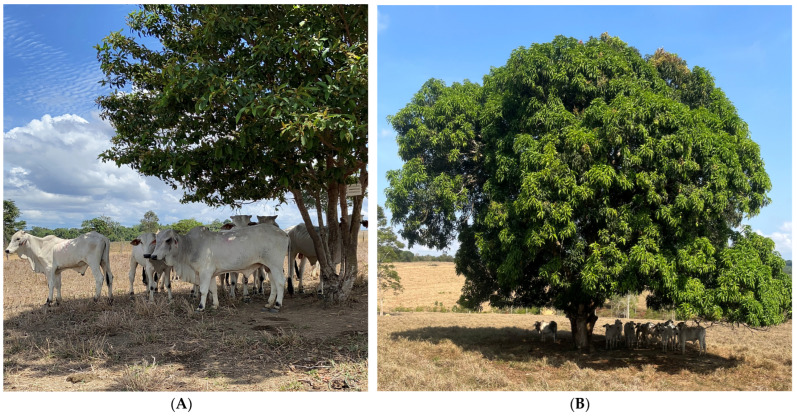
Cattle in shaded areas (**A**,**B**).

The fact that trees promote well-being and, consequently, shelter animals in periods of greater solar radiation intensity was evidenced in this study, as observed in Figure 7A,B.

Climate changes resulting from climatic events such as *El Niño* and their impacts on the pasture environment in the Amazon region, as observed in this study, serve as a subsidy for further research on the effects of changes associated with temperature and humidity on pasture productivity, the importance of shading and bathing areas during the hottest periods, as well as sustainable measures to reduce the impacts of climate change on the nutritional quality of forage and consequently on the diet of animals, in addition to developing sustainable strategies for the region of this study.

## 5. Conclusions

Under the conditions in which temperatures were highest during August and September, it was possible to conclude that the effects of *El Niño* in the Eastern Amazon, Brazil, were as follows:

- The full sun pasture area showed higher temperatures than the shaded area with the leafiest native species in the pasture, the Amazonian Brazil nut tree (*Bertholletia excelsa*), reinforcing the importance of maintaining the tree component in the region’s pastures. The leftover effect is diagnosed by the darker thermal patterns, which indicate lower temperatures, expressing the effectiveness of the shade provided by the trees in reducing heat and, consequently, increasing animal comfort.
- The analysis of average temperatures in native trees in the Amazon region, compared to the temperatures recorded in grasses and on the ground, underlines the ability of trees to create environments with higher environmental quality for cattle with light coats. The thermal difference between the native trees and their surroundings suggests a cooling effect provided by the trees, reinforcing their positive influence on thermal comfort in the area studied.
- It was possible to observe the numerous advantages of using infrared thermography to define efficient thermal patterns in the Eastern Amazon. These results corroborate the importance of the presence of trees in pasture management, not only to influence thermal conditions, but also to provide shade for animals, promote animal welfare and contribute to mitigating heat stress.

## Figures and Tables

**Table 1:** Descriptive data of the thermal targets analyzed in the different production systems in the Eastern Amazon.

Variables	Systems							
	SH				SN			
	T1	T2	T3	T4	T1	T2	T3	T4
Sample size	10	10	10	10	10	10	10	10
Minimum	36.1	36.2	34.8	32.7	38.9	36.2	34.3	32.7
Maximum	40.9	37.3	34.6	33.7	40.9	37.3	34.6	33.9
Total Amplitude	4.8	1.0	0.3	0.9	2.0	1.09	0.3	0.9
Median	39.29	36.4	34.5	33.3	39.5	36.4	34.5	33.3
First Quartile (25%)	39.1	36.2	34.4	33.1	39.1	36.2	34.4	33.1
Third Quartile (75%)	40.5	36.8	34.6	33.5	40.5	36.8	34.6	33.5
Interquartile deviation	1.4	0.5	0.1	0.3	1.3	0.5	0.1	0.3
Arithmetic average	39.4	36.5	34.5	33.2	39.7	36.5	34.5	33.2
Variance	1.9	0.1	0.01	0.8	0.6	0.1	0.01	0.08
Standard deviation	1.39	0.7	0.3	0.1	0.2	0.36	0.1	0.29
Standard Error	0.4	0.2	0.1	0.03	0.09	0.1	0.03	0.09
Coefficient of variation	3.55	2.0	0.9	0.2	0.8	0.9	0.2	0.8
Asymmetry	−1.32	0.6	0.9	0.1	−0.4	0.9	0.1	−0.4
Kurtosis	2.9	−1.4	0.1	−1.1	−0.4	0.1	−1.1	−0.4

Note: SH = Shadow, SN = Sun; T1 = white, T2 = red, T3 = yellow, T4 = green.

**Table 2:** Estimated means and standard deviations for temperature in each combination of system levels and treatments.

	SH		SN		Total	
	Average	SD	Average	SD	Average	SD
T1	36.472 a	0.255	39.796 a	0.798	38.134	1.800
T2	35.592 b	0.141	36.572 b	0.360	36.082	0.569
T3	35.005 c	0.040	34.534 c	0.101	34.769	0.253
T4	34.782 c	0.050	33.296 d	0.297	34.039	0.79
Total	35.463	0.677	36.049	2.530	35.756	1.863

Note: Different letters in the column indicate statistical differences (*p* < 0.05). SH = Shadow, SN = Sun. SD = standard deviations. T1 = white, T2 = red, T3 = yellow, T4 = green.

## Data Availability

The datasets presented in this article are not readily available because the data are part of an ongoing study. Requests for access to the datasets should be directed to welligton.medvet@gmail.com.
